# Not Only COVID-19: Involvement of Multiple Chemosensory Systems in Human Diseases

**DOI:** 10.3389/fncir.2022.862005

**Published:** 2022-04-25

**Authors:** Antonio Caretta, Carla Mucignat-Caretta

**Affiliations:** ^1^National Institute for Biostructures and Biosystems (NIBB), Rome, Italy; ^2^Department of Food and Drug Science, University of Parma, Parma, Italy; ^3^Department of Molecular Medicine, University of Padova, Padua, Italy

**Keywords:** chemosensation, olfaction, taste, chemesthesis, carotid bodies, pulmonary neuroendocrine cells, enterochromaffin cells, solitary chemoreceptor cells

## Abstract

Chemosensory systems are deemed marginal in human pathology. In appraising their role, we aim at suggesting a paradigm shift based on the available clinical and experimental data that will be discussed. Taste and olfaction are polymodal sensory systems, providing inputs to many brain structures that regulate crucial visceral functions, including metabolism but also endocrine, cardiovascular, respiratory, and immune systems. Moreover, other visceral chemosensory systems monitor different essential chemical parameters of “milieu intérieur,” transmitting their data to the brain areas receiving taste and olfactory inputs; hence, they participate in regulating the same vital functions. These chemosensory cells share many molecular features with olfactory or taste receptor cells, thus they may be affected by the same pathological events. In most COVID-19 patients, taste and olfaction are disturbed. This may represent only a small portion of a broadly diffuse chemosensory incapacitation. Indeed, many COVID-19 peculiar symptoms may be explained by the impairment of visceral chemosensory systems, for example, silent hypoxia, diarrhea, and the “cytokine storm”. Dysregulation of chemosensory systems may underlie the much higher mortality rate of COVID-19 Acute Respiratory Distress Syndrome (ARDS) compared to ARDSs of different origins. In chronic non-infectious diseases like hypertension, diabetes, or cancer, the impairment of taste and/or olfaction has been consistently reported. This may signal diffuse chemosensory failure, possibly worsening the prognosis of these patients. Incapacitation of one or few chemosensory systems has negligible effects on survival under ordinary life conditions but, under stress, like metabolic imbalance or COVID-19 pneumonia, the impairment of multiple chemosensory systems may lead to dire consequences during the course of the disease.

## Introduction

The ongoing COVID-19 pandemic has aroused some interest in taste and olfaction but even so, anosmia and dysgeusia are considered curious symptoms that at best may help in early differential diagnosis from benign respiratory tract infections (Menni et al., 2020) and, hopefully not, as a possible sign of subsequent central nervous system involvement, related to the so-called Long-COVID (Yong, 2021).

Chemical senses are commonly considered little more than evolutionary relics in humans. In everyday life, taste and olfaction are deemed relatively unimportant for many persons. As a matter of fact, often the impairment of olfaction was not even perceived by patients themselves (Croy et al., 2012), at least in pre-COVID-19 era. Olfaction or taste impairments, which may be quantitative or qualitative (Ercoli et al., 2021) are usually considered only with respect to the conscious awareness of these sensory inputs. Anyhow, these impairments may have important consequences on nutrition, social behavior, and mood (Croy et al., 2010, 2014a), as well as on work and quality of life (Brämerson et al., 2007). Actually, all the cells in our body may detect the presence of different molecules in the surrounding environment, yet only a few can use the detection of some chemicals to inform the central nervous system for organizing adaptive nervous or neuroendocrine responses that may impact the whole body. In addition to olfaction and taste, other cells monitor a wide range of chemicals inside our body: carotid bodies, solitary chemoreceptor cells, pulmonary neuroendocrine cells, and enterochromaffin cells. Furthermore, other ill-defined chemosensory cells are scattered throughout our body. Of all these inputs, we are rarely aware. In this review, we will briefly explore all these chemoreceptor systems and highlight their interconnections under physiological and pathological states. The following sections will introduce the chemical senses, their central connections and describe their involvement in human pathophysiology with the aim to highlight the intermingled contribution of different chemical receptors to health and homeostasis maintenance.

### Olfaction

Olfaction is probably the most intensively studied chemosensory system. Odorant molecules may access the olfactory mucosa, which hosts the olfactory receptor neurons, directly from the nostrils leading to the orthonasal perception, or from the buccal cavity, called the retronasal perception (Rombaux et al., 2009). An impressive fraction of the mammalian genome, comprising more than 300 genes in humans and even more in other mammals (Olender et al., 2008), is dedicated to code for olfactory receptor proteins (Buck and Axel, 1991), the canonical olfactory receptors, apparently responsible for recognizing odors. They are seven-transmembrane G-protein-coupled receptors (GPCRs), monoallelicaly expressed in large amounts in olfactory neurons (Table 1). Notably, they are expressed also in many other tissues (see Maßberg and Hatt, 2018) and are deeply involved, among other functions, in angiogenesis and vascular tone modulation in the blood vessels (Dalesio et al., 2018), and in regulating lipid and glucose metabolism (Zhang et al., 2021). In addition, other receptors, although mainly expressed in other tissues, are present to a lesser extent also in olfactory sensory neurons. In humans, there are six genes encoding for the trace amine-associated receptors (TAARs), five of which are expressed in olfactory mucosa. They are seven-transmembrane domain GPCRs (Fleischer et al., 2009) involved in recognizing neurotransmitters and microbial by-products for regulating the release of nutrient-related hormones and in the development of immune cells (Gainetdinov et al., 2018). TAARs are also expressed in the olfactory bulb, and they modulate limbic brain areas involved in the emotional control of anxiety and depressive behaviors (Espinoza et al., 2020). The 7-transmembrane GPCRs formyl-peptide receptors are encoded by three genes in humans. In mice olfactory mucosa, they switched from an immune role to external molecules sensing, yet they still may trigger immune responses (Dietschi et al., 2017). The olfactory epithelium also hosts microvillous cells, which have been suggested to modulate local responses (Hansen and Finger, 2008). They express the TrpM5 receptor, a calcium-activated voltage- and temperature-dependent channel for monovalent cation influx, which serves as downstream effector in bitter, sweet, and umami transduction both in taste and solitary chemoreceptor cells (see below; Burman and Kaji, 2021). Other receptors are present in rodents, but they are either absent or debated in humans, and these include the membrane guanylyl cyclase-D in olfactory and septal organ neurons and the seven transmembrane GPCRs vomeronasal receptors type 1 and 2 (Fleischer et al., 2009), not reviewed here.

**TABLE 1:** Main receptor types expressed in the different peripheral chemosensory structures.

Receptor	Olfactory mucosa	Trigeminal nerve	Taste buds	Carotid bodies	Aortic bodies	Solitary chemoreceptor cells	Entero-chromaffin cells	Pulmonary neuroendocrine cells
**GPCR**								
Olfactory receptors	X		X	X			X	X
Trace amine-associated receptors	X						X	
Formyl-peptide receptors	X							
T1R1-T1R3 umami taste receptor			X				X	
T1R2-T1R3 sweet taste receptor			X			X		
T2R bitter taste receptor			X			X	X	
GPR* fatty acids receptors			X				X	
**TRP**								
TRPM5	X	X	X			X		
TRPM8		X	X					
TRPA1		X					X	
TRPV1		X	X					
TRPV3		X						
Trpc5								X
***Ionic channels***								
Acid-sensing Na^+^ channels (ASICs)		X						
Two-pore domain K^+^ channels (KCNK)		X		X				
K_*ATP*_			X					
OTOP1 proton channel - sour taste receptor			X					
Epithelial Sodium channels (ENaC) -salty taste			X					
P2X ATP receptors			X	X	X			X
NADPH oxidase-K^+^ channel								X

*See text for details and references. GPCR, G-protein coupled receptors; TRP, transient receptor potential channels; X, present.*

*Several receptors, see text.

Moreover, receptors for many hormones, pertaining to metabolism homeostasis, are present in olfactory mucosa, including receptors for insulin, leptin, orexin, cholecystokinin, adiponectin, NPY, and ghrelin, in addition to glucose transporters and fatty acid sensors like CD36 (Palouzier-Paulignan et al., 2012; Julliard et al., 2017). Also, the olfactory system is modulated by the internal state (McIntyre et al., 2017). Hence, it makes sense to consider the main olfactory system, not as a specific sensory system able to detect only external molecules (odors), but rather as a polymodal sensory system activated and modulated both by external and internal chemical signals.

### Trigeminal Chemesthesis

Besides olfactory receptors, the nose and the mouth host many trigeminal terminals, which are present also in the eye (not reviewed here) and are responsible for trigeminal “chemesthesis,” presumably a mix of pain, tingling, thermal, numbing, and other sensations arising from chemical receptors (Hummel and Frasnelli, 2019). These terminals are stimulated by CO_2_, menthol, capsaicin, eucalyptol, and a variety of other stimuli (Konstantinidis et al., 2010; Hummel and Frasnelli, 2019). The receptors for irritant chemical stimuli have been identified in the trigeminal terminals as transient receptor potential (TRP) channels and acid-sensing ion channels (ASICs), which are heterotrimeric and permeable to sodium, and two-pore potassium channels (KCNK; Simons et al., 2019). Trigeminal afferents express the following TRP channels, which are six-transmembrane domains homotetramer cation channels: TRPA1, broadly tuned to give pungent sensation and with multiple regulations; TRPV1, which mediates capsaicin hot and acid-sensing; TRPV3, which mediates thymol, eugenol, and fatty acids metabolites; and TRPM8, which mediates the freshness of menthol, while TRPM5 is expressed in trigeminal ganglia (Vandewauw et al., 2013). The first two may also modulate bitter, sour, and salt taste perception by releasing neuromodualtors like CGRP and substance P (Hummel and Frasnelli, 2019; Rhyu et al., 2021). The trigeminal stimuli may interact with olfaction peripherally (Tremblay and Frasnelli, 2018), modulate olfactory bulb activity in the rat (Schaefer et al., 2002), and greatly contribute to the food flavor perception, besides eliciting protective reflexes like sneezing and coughing (Klein, 2019).

### Taste

Strictly speaking, taste depends on the functioning of taste buds present in the oral cavity, mainly on the tongue. The sensory cells within these structures express membrane receptors responsible for recognizing the five traditional sensory modalities of taste: bitter, sweet, sour, salty, and umami (Kinnamon and Finger, 2019). Sweet and umami taste receptors are heterotrimeric GPCRs, both are dimers composed of the T1R3 subunit coupled to the T1R1 for umami and T1R2 for sweet receptors (Kochem, 2017). For sweet taste, a parallel route involving glucose transporters and K_*ATP*_ channels triggering calcium and GLP-1 signaling has been proposed (Takai et al., 2015; von Molitor et al., 2020). There are at least 25 bitter taste receptors (T2Rs) in humans pertaining to the 7-transmembrane GPCR T2R family (Behrens et al., 2007). The sour taste receptor has been recently identified in mice as OTOP1, a proton channel with 12 transmembrane domains, assigning an ancillary role to the amiloride-sensitive sodium channel ASIC, the hyperpolarization-activated and cyclic-nucleotide gated HCN4, and PKD2L1, a TRP channel (Turner and Liman, 2022). Salty taste in humans, like in rodents, appears mainly mediated by the heterotrimeric (α, β, and γ) epithelial sodium channel (ENaC), with humans expressing also a fourth subunit (δ) localized to the taste-pore region of taste cells (Bigiani, 2020).

As is the case for canonical olfactory receptors, also taste receptors (mainly bitter and sweet receptors) are expressed in many other cell types outside the mouth and in turn may modulate the release of different hormones (Behrens and Meyerhof, 2019). All these cell types, including taste receptor cells and taste receptor-expressing cells like tuft, brush, or solitary chemoreceptor cells in different tissues, may have a common phylogenetic origin and may serve defensive or digestive functions (Sbarbati and Osculati, 2005). Bitter taste receptors are expressed in the lung-smooth muscle with a bronchodilation function (An and Liggett, 2018). Also, bitter taste receptors are found in testis, bladder, heart, kidney, brain, airway epithelial, immune, and smooth muscle cells, serving different functions according to the molecular machinery and possible neural connections of the different cell types (Lu et al., 2017; Martens et al., 2021).

In addition to taste receptors, also other types of receptors are expressed in sensory cells of taste buds: a high number of molecules involved in various immune mechanisms, especially of the innate system, are present, including Toll-like receptors (Wang et al., 2009), cytokines, and their receptors (Feng et al., 2014). Recently, taste cells were also found to express olfactory receptors (Malik et al., 2019). Moreover, as is the case for olfactory neurons, receptors for many hormones and neurotransmitters are also expressed on taste bud cells (Calvo and Egan, 2015). Lastly, different receptors of the TRP family are involved in different sensory mechanisms, like thermal, mechanical, and pain sensations, and also in taste. Notably, genomic polymorphism of TRPV1 has been associated with pain and salty taste sensitivity (Aroke et al., 2020).

### Other Chemoreceptors: Carotid and Aortic Bodies, Solitary Chemoreceptor Cells, Pulmonary Neuroendocrine Cells, and Enterochromaffin Cells

Other chemosensory systems are present within our body, but at variance with olfaction and taste, they convey information on internal molecules. While providing fundamental inputs for homeostasis, they rarely give rise to conscious perception.

#### Carotid and Aortic Bodies

Carotid bodies are positioned close to carotid bifurcation and have multiple functions. Their structure closely resembles that of taste buds (Gonzalez et al., 2014), and similarly, they use purinergic transmission between sensory cells and nerve terminals (Finger et al., 2005). Carotid bodies are innervated by the glossopharyngeal (Hering’s) nerve expressing P2X purinergic receptors at complex quadripartite synapses. These sense ATP released from chemosensory cells, which are excited by the inhibition of resting potassium currents via TASK two-pore domain potassium channels (Buckler, 2007; Piskuric and Nurse, 2013). Besides pannexin-1, different connexins functional hemichannels are present in the carotid body sensory cells and nerve terminals, Cx43 and Cx36, respectively, and may contribute to cell coupling under resting state, which is relieved upon chemosensory stimulation (Reyes et al., 2014). The first function that was described for carotid bodies was their ability to measure oxygen partial pressure in plasma and, to a lesser extent, plasma CO_2_ and pH. These incoming data allow the regulation of gases and acid–base balance in the blood, and ventilation in a reflex way, as a consequence of stressor stimuli like sleep apneas, exposure to high-altitude environment, but also in lung diseases like asthma and COPD (Whipp, 1994). Actually, the activation of carotid bodies induces a reflex bronchoconstriction (Nadel and Widdicombe, 1962), claiming for carotid body denervation as a treatment for asthma and COPD (Winter and Whipp, 2004). Moreover, carotid bodies orchestrate the responses to physical exercise, a common physiological stressor, allowing the estimation of functional capacity and hence predicting the clinical responses also to non-physiological stressors like surgery (Levett et al., 2018).

Glucose sensing, which serves a vital homeostatic function, is under the control of multiple central (ventromedial hypothalamus and several areas in the pons and medulla) and peripheral chemosensors located in the oral cavity, gastrointestinal tract, portal vein, and curiously, in the carotid bodies (Donovan and Watts, 2014). Carotid bodies are indeed sensitive to glucose (Pardal and López-Barneo, 2002) and metabolic signals and also to inflammatory signals since insulin, leptin, and pro-inflammatory cytokines activate the carotid bodies, inducing a sympathetic overactivation that leads to glucose intolerance and insulin insensitivity (Sacramento et al., 2020). The carotid body-induced sympathetic overactivation may thus underlie metabolic syndrome and systemic hypertension (Kim and Polotsky, 2020) and justify carotid body denervation or pharmacological targeting as possible therapies (Lastra et al., 2014; Pijacka et al., 2016; Sacramento et al., 2017). These, however, are not without risk, given the relevance of carotid bodies in the response to acute hypoxia and hypercapnia in particular in hypertensive subjects (Conde, 2018). Hence, carotid body impairment is involved to various extents in the pathophysiology not only of respiratory dysfunction but also of hypertension, insulin resistance, and metabolic syndrome. The molecular machinery subtending these abilities is still under investigation, anyhow carotid bodies are known to express canonical olfactory receptors like Olfr78 (the mouse ortholog of human OR51E2) that senses lactate produced under hypoxia (Chang A. J. et al., 2015) and ion channels, like the ATP-sensitive P2X2 ionotropic receptor, which are similar to those of taste nerve processes (Rong et al., 2003; Finger, 2005; Yang et al., 2012), depicting similar processes for transmission of information (Kinnamon and Finger, 2013).

At variance with carotid bodies, which are clumped at a single site and are sensitive to arterial oxygen partial pressure, aortic bodies are patches of chemosensory cells innervated by the vagus nerve, distributed along the aortic arch. Their function is less explored in humans; in experimental animals they are sensitive to hemoglobin oxygen saturation (Piskuric et al., 2014). Red blood cells release ATP via pannexin-I channels in response to the conformational change in desaturated hemoglobin, and ATP, in turn, activates P2X receptors on aortic bodies’ nerve terminals to enhance the response of local chemosensory cells, which are supposed to share the same transduction mechanism as carotid bodies (Piskuric and Nurse, 2013). Aortic bodies mainly serve, together with similar chemosensory cells sparsely located in the thorax and abdomen, to regulate circulation (Piskuric and Nurse, 2013) by triggering reflexes in response to hypoxiemia, in particular when carotid bodies’ function is blunted or absent (Prabhakar and Peng, 2004). Actually, both these systems sense different features of oxygen shortage and foster the relevant functional response, which are enhanced respiratory drive via carotid bodies for decreased oxygen partial pressure and increased cardiovascular activation for decreased hemoglobin saturation, thus paving the way for addictive responses.

#### Solitary Chemosensory Cells

Solitary chemosensory cells (Finger et al., 2003), also known as brush cells in airways and tuft cells in the gastrointestinal tract, are present on most mucosal surfaces including nasal, oral, respiratory, and intestinal mucosa (Sbarbati and Osculati, 2005). Noteworthy, many solitary chemosensory cells are interspersed in the nasal mucosa. These cells express canonical T2R bitter taste receptors and transduction pathways, sense bitter molecules including those originated by bacteria, stimulate the trigeminal terminals with acetylcholine, and participate in regulating immune responses (Carey et al., 2016), although presumably they are involved also in other functions. They are polymodal chemosensors for irritants and bacterial signals that release acetylcholine and are also involved in regulating mucosal innate immune response (Tizzano et al., 2010; Saunders et al., 2014). These cells form a heterogeneous population since sometimes they may co-express T2R and T1R taste receptors, while in other cases cells do not appear innervated (Tizzano et al., 2010).

In the lower airways, tuft cells release acetylcholine when stimulated by bacterial compounds and they modulate ciliary beating, while the function of tuft cells in the stomach and the urethra appears less clear, even if their number is associated with microbial colonization and appears to drive the antimicrobial response (Schneider, 2021). In the small intestine, tuft cells express both T1R and T2R (O’Leary et al., 2019). They sense a variety of molecules and are closely linked to type 2 immunity, triggering aversive responses through immune and neuronal cells (Schneider et al., 2019).

#### Enterochromaffin Cells and Pulmonary Neuroendocrine Cells

At the mucosal interface between the internal and external environment, similar cells, namely enterochromaffin cells in the gut and pulmonary neuroendocrine cells in the lungs, monitor the chemical features of the external environment.

Pulmonary neuroendocrine cells are a small percentage (less than 1%) of pulmonary epithelial cells, innervated by the vagus nerve (Noguchi et al., 2020). They sense oxygen through a NADPH oxidase coupled to a potassium channel sensitive to oxygen. They may sense multiple chemical parameters from hypoxia to air pollutants in bronchial air (for example nicotine sensing through nicotinic Ach receptors), secrete serotonin, other peptides, and also ATP, which acts on autoreceptors and nerve terminals’ P2X receptors, and are involved in regulating Type 2 immune response (Noguchi et al., 2020). They amplify allergic asthma responses (Sui et al., 2018) via a neuroimmune mechanism (Pavón-Romero et al., 2021), yet mitigate hypoxic injury (Shivaraju et al., 2021). Besides, they are also mechanosensitive through the cation channels Piezo2 and Trpc5 (Noguchi et al., 2020). They appear overactive in patients suffering from lung diseases, including asthma, COPD, and bronchopulmonary dysplasia (Noguchi et al., 2020), and contribute to altered sensitivity toward volatile molecules (Gu et al., 2014). In the respiratory system, they are also involved in regulating inflammatory response (Branchfield et al., 2016), lung blood flow (Fu et al., 2002), and bronchial muscle tone (Skogvall et al., 1999).

Both the enterochromaffin cells and pulmonary neuroendocrine cells, as isolated cells or clustered at airway bifurcations in neuroepithelial bodies, whose anatomical structure closely resembles that of carotid bodies and taste buds (Lauweryns and Cokelaere, 1973), express GPCRs including canonical olfactory receptors; enterochromaffin cells also express bitter taste T2R receptors and trace amine-associated receptors (TAR1; Kidd et al., 2008; Gu et al., 2014; Bellono et al., 2017). Interestingly, the stimulation with an olfactory receptor ligand triggers serotonin release in the colon and modulates anion secretion and epithelial permeability (Kaji et al., 2011).

Enterochromaffin cells also express TRPA1, the alpha2A-adrenoreceptor, SNAT1/2 glutamine transporters, GLUT and SGLT glucose transporters, ABST bile salt transporters, and toll-like receptors (Kidd et al., 2008; Bellono et al., 2017; Wang H. et al., 2019). They are by far the major source of serotonin in mammals (Erspamer, 1937), and their afferent signals are carried mainly by vagus nerve fibers (Hagbom et al., 2011). They release serotonin in response to glucose and evoke a reflex diminution in gastric motility and subsequent emptying (Raybould et al., 2003). Enterochromaffin cells can sense multiple metabolic and homeostatic parameters of the intestinal lumen, for example, glucose and free fatty acids from microbiota (Reigstad et al., 2015) and relay them to the nerve cells (Bellono et al., 2017). In this way, they can monitor energy substrates’ availability in the intestinal lumen through glucose and free fatty acids receptors, activate afferent fibers, and regulate storage and the use of nutrients; hence they take part in controlling body metabolism and energy expenditure. These cells can detect luminal antigens and modulate the immune response to intestinal microbiota by activating the mucosal immune system. Moreover, they are sensitive to mechanical stimuli and hence regulate gastrointestinal motility (Martin et al., 2017). Enterochromaffin cells are a subset of enteroendocrine cells (Gunawardene et al., 2011), which sense long-chain fatty acids through CD36, GPR40, and GPR120; short-chain fatty acids through GPR41 and GPR43; endogenous lipid metabolites with GPR119; glucose through SGLT1; aminoacids through umami taste receptor, CaSR, and GPRC6A; and peptides through PepT1, leading to GLP1 and cholecystokinin release (Raka et al., 2019; Burman and Kaji, 2021; Duca et al., 2021). They are innervated by the vagus nerve (Kaelberer et al., 2018), depicting a neural pathway for direct nutrient sensing.

## Chemosensory Central Connections

Afferent pathways from the peripheral chemosensory cells to the central nervous system can be structured in two sections, one entering from the brainstem and one from the olfactory bulb, but notably, inside the brain, they largely converge on the same areas (see Table 2 for a summary).

**TABLE 2:** Summary of the main projection areas of the different peripheral chemosensory systems.

	Primary afferents	First relay station within the brain	Subcortical projection areas	Cortical integrative area
Main Olfactory epithelium	Olfactory nerve	Main olfactory bulb	Amygdala, hypothalamus	Olfactory cortex Orbitofrontal cortex
Trigeminal nerve	Trigeminal nerve	Nucleus of the solitary tract, trigeminal spinal nucleus	Hypothalamus	Gustatory cortex Orbitofrontal, cortex
Taste buds	Facial, glossopharyngeal, and vagus nerves	Nucleus of the solitary tract, trigeminal spinal nucleus	Hypothalamus	Gustatory cortex Orbitofrontal cortex
Carotid bodies	Glossopharyngeal nerve	Nucleus of the solitary tract	Retrotrapezoid nucleus - Brainstem respiratory centers	*
Aortic bodies	Vagus nerve	Nucleus of the solitary tract	Brainstem cardiovascular control centers	*
Solitary chemosensory cells	Trigeminal nerve (lung)	Nucleus of the solitary tract	Brainstem nuclei, hypothalamus	*

*Integration can be achieved through additional passages so that stimulation can in some cases become conscious and lead to conscious responses. See text for details and references.

Chemosensory systems, including taste and olfaction, have many intricated subcortical connections also in the primate brain and, regardless of sensory information they convey, they are in a position to take part in shaping the integrated responses to external and internal stress conditions, under almost every instance. These responses involve cardiovascular, respiratory, endocrine, and immunologic functions that are essential for coping effectively with stress, and hence for survival. A brief outline of chemosensory central nervous pathways will highlight their complex connections.

Humans have higher olfactory thresholds than macrosmatic mammals, although not so poor as commonly thought. Yet, when it comes to olfactory pathways, human subcortical connections are extensively developed, similar to other mammals. In other mammals, different sensory organs are hosted in the nasal cavity, including vomeronasal, septal organ, and Grueneberg ganglion, all projecting to the (main or accessory) olfactory bulbs (Weiler and Farbman, 2003; Matsuo et al., 2012; Mucignat-Caretta, 2021), while in humans, olfactory fibers originate almost exclusively from main olfactory mucosa and run through the first cranial nerve, the olfactory nerve, and contact olfactory bulb mitral cells, the main collector of afferents. From here, olfactory projections radiate mainly, yet not exclusively, to three amygdala subregions, possibly involved in the setup of motor response to olfactory stimuli (medial amygdala), reward and memory processes (cortical amygdala), and multisensory integration (periamygdaloid complex; Noto et al., 2021). Notably, amygdala expresses the acid-sensing ion channel ASIC1a, which centrally senses hypercapnia and acidosis and drives the subsequent fear responses in mice (Ziemann et al., 2009), while the human hortolog ACCN2 is associated with amygdala structure and function in panic disorders (Smoller et al., 2014). The human orbitofrontal cortex is the final recipient of sensory information and is involved in reward and emotional value of sensory stimuli, including olfaction and taste (Rolls, 2021), and decision-making in olfactory-driven food intake (Seabrook and Borgland, 2020). Other projections reach the olfactory nucleus, taenia tecta, entorhinal and piriform cortex, the so-called olfactory cortices, and olfactory tubercle, which appears to modulate odor-guided eating (Murata, 2020). Interestingly, also trigeminal stimuli can activate the piriform cortex, converging on the same chemosensory integrative center after entering the brain from the hindbrain (Hummel and Frasnelli, 2019). Moreover, all these areas receive modulatory dopaminergic inputs from the midbrain ventral tegmental area involved in reward (Oades and Halliday, 1987; Deutch et al., 1988). From the entorhinal cortex, olfactory information then reaches the hippocampus, while subsequent projections from the amygdala run to the hypothalamus (premammillary nuclei, medial preoptic area, and ventromedial nucleus) and bed nuclei of stria terminalis, thus involving, according to old terminology, the limbic system, well known for controlling emotional behavior. Interestingly, the bed nuclei of the stria terminalis are involved in anxiety behavior triggered by hypercapnia and acidosis (Taugher et al., 2014). The bed nuclei of stria terminalis and hypothalamus are in turn both connected to many other brain areas, including the brainstem nuclei (Mucignat-Caretta, 2021), and are involved in linking inflammation to complex homeostatic behaviors, like feeding and its dysregulation (Guillemot-Legris and Muccioli, 2017; Wang Y. et al., 2019). The hypothalamus appears thus as a chemosensory hub that regulates not only emotional and social, often competing, behavior but also visceral homeostatic functions like glycemia, appetite, energy expenditure, blood pressure, heart function, respiration, and immune system through neurohormonal and sympathetic activation to downstream hindbrain targets (Henderson and Macefield, 2021; Khodai and Luckman, 2021; Nakamura et al., 2022).

At variance to olfaction, most other chemosensory afferent fibers enter into the brainstem through the 5th (trigeminus), 7th (facial), 9th (glossopharyngeal), and 10th (vagus) cranial nerves. From the middle of the XIX century, brainstem is known to regulate mammalian metabolism, after Claude Bernard’s so-called piqûre diabétique, actually transient hyperglycaemia and glycosuria, triggered by the puncture of the floor of the fourth ventricle (Li, 1952; Feldberg et al., 1985); hence it is not surprising that the center that orchestrates visceral output according to sensory inputs resides in the brainstem. This center is the nucleus of the solitary tract.

Gustatory afferents converge in a rostro-caudal topographic way through facial, glossopharyngeal, and vagus nerves on the nucleus of the solitary tract and then relay (in rodents) to the pontine parabrachial nuclei, and the motor nuclei of cranial nerves to mediate taste-induced reflex responses (Rolls, 2019). Noteworthy, there is a large anatomical overlap between visceral chemosensory afferent fibers and taste afferents in peripheral nerves. Most afferent fibers from the carotid body and tongue posterior taste bud are already intermixed, both running through glossopharyngeal nerve and petrosal ganglion (Leonard et al., 2018). Afferent vagal fibers carry sensory information from pulmonary neuroendocrine cells (van Lommel et al., 1998) and express the purinergic receptor P2RY1, while other vagal sensory afferents from the lung express both NPY2 receptor and the capsaicin receptor TRPV1 (Chang R. B. et al., 2015). Afferent vagal fibers also carry information from some taste buds in the palate and epiglottis, solitary chemosensory cells in the lower airways (Krasteva et al., 2011), enterochromaffin cells (Hagbom et al., 2011), and glucose-sensing neurons (Browning, 2010; Grabauskas et al., 2010), and also from the aortic bodies (Piskuric and Nurse, 2013). Some afferent vagal fibers from the gut express olfactory receptors and may participate in the control of feeding with mechanosensitive vagal afferents (Bai et al., 2019). Notably, astrocytes within the nucleus of the solitary tract respond to glucoprivation and participate in increasing the gastrointestinal motility (McDougal et al., 2013). Afferent trigeminal fibers carry information from oral and nasal solitary chemosensory cells (Carey et al., 2016). Of note, the trigeminal spinal nucleus receives inputs from cranial nerves 5, 7, 9, and 10.

Then, from the nucleus of the solitary tract, through the ventroposteromedial (VPM) nucleus of the thalamus, taste inputs reach the gustatory cortex, namely the anterior insula in the temporal lobe and the frontal operculum (Rolls, 2019), and terminate in the orbitofrontal cortex, which integrates multiple sensory inputs, including olfactory (see above, Rolls, 2021), and ultimately affects food intake and visceral modulation, besides memory (Vincis and Fontanini, 2019). However, a second bidirectional connection originates from the nucleus of the solitary tract to subcortical targets: the lateral hypothalamus, central nuclei of amygdala, and bed nuclei of the stria terminalis, and areas involved in feeding, autonomic modulation and emotional control, as mentioned above.

Also, the carotid bodies project to the nucleus of the solitary tract, which is bidirectionally connected with the retrotrapezoid nucleus in the rostroventral medulla oblongata: this nucleus also contains central chemoreceptors to detect local hypercapnia or acidification, including the proton-modulated TASK-2 potassium channels and the proton-activated GPCR GPR4 that drive the respiratory reflexes. Also, in these responses astrocytes are involved to modulate neuronal activation (Guyenet et al., 2016). Connexin 26 hemichannels are present in astrocytes within this nucleus: they are gated by CO_2_ and participate in the centrally-driven ventilatory response to hypercapnia (van de Wiel et al., 2020). Electrical coupling driven by connexins was also found in the nucleus of the solitary tract under hypercapnic acidosis (Reyes et al., 2014).

The nucleus of the solitary tract, which is embedded in brainstem reticular formation, receives an astonishing number of different, not only chemosensory, afferents from basically the entire body through facial, glossopharyngeal, and vagus nerves. From a physiological point of view, it is a crucial hub, integrating incoming sensory signals with hypothalamic outflow, and being potentially connected to the whole brain and body, it is the ideal anatomical center to drive autonomic, neurohormonal, and behavioral responses to stress (Holt, 2021), both acute and chronic (Herman, 2018).

## Chemosensation in Human Pathophysiology

Dysfunction of carotid body/glossopharyngeal nerve has also been postulated in the potential pathogenesis of non-respiratory diseases like metabolic syndrome-type 2 diabetes and hypertension (Kim and Polotsky, 2020; Sacramento et al., 2020). In the past, vagal dysfunction has been hypothesized in luetic pain (Mylona et al., 2010; Klein and Ridley, 2014), gastroduodenal ulcer (Szabo et al., 2016; Zalecki et al., 2020), and more recently in obesity-metabolic syndrome (Berthoud and Neuhuber, 2019), but on the whole, in clinical practice, little attention is currently paid to the involvement of chemosensory systems in these diseases. A possible explanation is that chemosensory systems are not considered essential for human life, and hence have a minor position in medical training. Impairments in taste and olfaction in some cases are not even perceived by the patients themselves and do not affect body survival. With the exception of a few professionals, loss of olfaction or taste is rated very low by health insurance companies also.

Notwithstanding their importance, bilateral therapeutic ablation of carotid bodies in humans leads to apparently negligible side effects (Iturriaga et al., 2021). Therapeutic vagotomies have been performed in humans with only minor side effects (Szabo et al., 2016). The same is true for afferent trigeminal signals generated in the oral and nasal chemoreceptors (Barham et al., 2013), and a perturbation of this pathway seems to bear minor consequences. In mice, ablation of pulmonary neuroendocrine cells is apparently harmless (Song et al., 2012). These puzzling data suggest that the lack of a single chemosensory input is dispensable, or at least may be compensated by other inputs.

## Olfaction and Taste on the Stage With COVID-19

Interest in taste and olfaction as diagnostic and/or prognostic tools has been fuelled by the recent COVID-19 pandemic, during which many persons developed chemosensory symptoms. The loss of sense of smell apparently had a prevalence of 47%, ranging from 11 to 84%, due to differences in symptom detection (for a discussion see Karamali et al., 2022), with psychophysical testing being more reliable than self-report (Bordin et al., 2021). The actual prevalence of these disorders is hardly estimated because patients may not report paucisymptomatic disease or may not be aware of their disturbances. Actually, psychophysical testing detects more subjects with impaired sensitivity than subjectively reported (Hummel and Podlesek, 2021). In addition, with the spreading of vaccination, a difference in prevalence between pandemic waves could not be attributed to differences in virus variants since the appearance of symptoms in vaccinated people may depend on their immunological state. Moreover, virus sequencing requires expansion in cell cultures to obtain enough viruses for sequencing, which may introduce variations in genomic sequence, an issue rarely taken into account in the literature (World Health Organization [WHO], 2021, p. 18). Another critical point is the process of recovery, which may benefit from olfactory training (Addison et al., 2021; Denis et al., 2021), yet may be not smooth, since some olfactory symptoms like parosmias and phantosmias may emerge well after the end of the infection, and in some cases, olfactory recovery does not take place (Cecchetto et al., 2021; Ohla et al., 2022). The different chemosensory systems (taste, olfaction, carotid body, solitary chemosensory cells, pulmonary neuroendocrine cells) share common molecular characteristics, including some receptors and integrative centers (see above). Hence, it is conceivable that, in addition to taste, olfaction, and trigeminal chemesthesis (Parma et al., 2020), other chemosensory systems may also be affected by SARS-CoV2 virus. As a matter of fact, the virus has been detected in autoptical carotid bodies of patients with COVID-19 (Lambermont et al., 2021). Notably, ACE2, TMPRSS2, and Neuropilin-1, the three entry sites for SARS-CoV2, have been recently found in the glossopharyngeal and vagus nerves near the medulla (Vitale-Cross et al., 2022). Apparently, SARS-CoV2 enters the sustentacular cells in olfactory mucosa and destroys it (Bryche et al., 2020), opening its way to the endothelial and nervous tissues up to the central nervous system (Meinhardt et al., 2021), even if the direct involvement of the olfactory nerve seems implausible (Butowt et al., 2021).

It can be suggested that some peculiar symptoms of COVID-19 patients may be explained by the impairment of chemosensory systems. The “silent hypoxia,” that is the absence of dyspnea in the presence of life-threatening hypoxemia, may depend on the damage of carotid bodies; moreover, the impairment of other oxygen sensor systems like the pulmonary neuroendocrine cells may be involved, causing a lack of hypoxic pulmonary vasoconstriction (Caretta and Mucignat-Caretta, 2021). The gastrointestinal symptoms of COVID-19 patients are related to an increase in serotonin levels (Ha et al., 2021; Jin et al., 2021), presumably dependent on the dysregulation of enterochromaffin cell function, which usually produces the vast majority, roughly 90%, of gut serotonin (Koopman et al., 2021). An increase in blood serotonin concentration, again linked to impairment of enterochromaffin cell function, is reported in inflammatory bowel disease (El-Salhy et al., 1997) and in rotavirus diarrhea (Hagbom et al., 2011).

Also, the so-called cytokine storm, that is the dysregulation of innate immune response leading to COVID-19 ARDS catastrophic outcome (Hue et al., 2020), may be related to the impairment of vagal immune reflexes (De Virgiliis and Di Giovanni, 2020). The brain fine-tunes the anti-inflammatory pathway via cholinergic vagal efferents, whose stimulation may prevent fatal endotoxemia (Tracey et al., 2001; Andersson, 2005; Chavan et al., 2017). This is in addition to the extensive regulation of respiration and lung defensive reflexes constantly operated via mechano- and chemosensory vagal inputs (Mazzone and Undem, 2016).

Lastly, solitary chemosensory cells (Saunders et al., 2014) and enterochromaffin-pulmonary neuroendocrine cells may be involved in the inflammatory pathway or the immune modulation (Koopman et al., 2021; Pavón-Romero et al., 2021).

## Chemosensory Disturbances in Human Pathology

Loss of taste and olfaction are easily and non-invasively tested *in vivo*. It is not specific of COVID-19 disease, since it may be caused by many different chemicals, infections, traumatic, or neurodegenerative processes. However, also during physiological aging, a decrease in chemosensory performance is well-documented (Landis et al., 2004; Seubert et al., 2017), and increasing age is associated with the rising prevalence of smell disturbances (Bhattacharyya and Kepnes, 2015). Notably, dysfunction of olfaction correlates with increased mortality and with patient frailty in the general population, possibly mediated by inflammation (Liu et al., 2019; Laudisio et al., 2019). Conversely, dysfunction of olfaction and/or taste has been reported in many diseases encompassing dysregulation of multiple visceral functions. In addition to head trauma (Howell et al., 2018), tumors affecting the olfactory areas and epilepsy of the temporal lobe affecting olfactory areas, for which olfactory involvement is apparent, given below is a non-exhaustive list of diseases and conditions in which an impairment in olfaction (most often), taste, or both, has been demonstrated in human patients (see Table 3):

**TABLE 3:** List of the diseases in which olfactory and/or taste impairments are most frequently detected (see text for additional information).

Process/organ	Disease	Relevant references
Physiological process	Aging	Landis et al., 2004; Bhattacharyya and Kepnes, 2015; Seubert et al., 2017
Poisoning (acute or chronic)	Peripheral or central degeneration	Genter and Doty, 2019
Infectious diseases	COVID-19	Karamali et al., 2022
	Different viruses, bacteria, fungi, and protozoa	Doty, 2019
Neurodegenerative diseases	Parkinson’s disease	Marin et al., 2018
	Multiple sclerosis	Fleiner et al., 2010; Goverover et al., 2020
	Alzheimer’s disease and dementia	Lang et al., 2006
Neuropsychiatric diseases	Psychosis	Catalan et al., 2021
	Anorexia nervosa	Mai et al., 2020
	Depression	Athanassi et al., 2021
Dysmetabolic diseases	Obesity	Holinski et al., 2015
	Metabolic syndrome-type 2 diabetes	Rasmussen et al., 2018; Zhang et al., 2019; Catamo et al., 2021; Faour et al., 2022
	Type 1 diabetes	Falkowski et al., 2020
	Cachexia	Hutton et al., 2007; Zhou et al., 2017
	Anorexia of elderly	Wysokiński et al., 2015
Cancer	Lung cancer	Spencer et al., 2021
	Metastatic cancer	Landis et al., 2004; Spotten et al., 2017
Autoimmune disorders	Fibromyalgia	Amital et al., 2014
	Systemic lupus erythematosus	Bombini et al., 2018
	Autoimmune involvement in neuropsychiatric disorders	Moscavitch et al., 2009
Cardiovascular diseases	Cardiovascular disease	Roh et al., 2021
	Coronary heart disease	Seubert et al., 2017
	Hypertension	Liu et al., 2018
Kidney diseases	Chronic kidney disease	Griep et al., 1997; Nigwekar et al., 2017; Robles-Osorio et al., 2020; Iacono et al., 2021
Liver diseases	Liver disease, cirrhosis	Landis et al., 2004; Heiser et al., 2018
Gastrointestinal disorders	inflammatory bowel diseases	Steinbach et al., 2013; Sikander et al., 2015
Lung diseases	COPD	Chapman-Novakofski et al., 1999
	Cystic fibrosis	Di Lullo et al., 2020

*Sporadic reports of chemosensory disturbances in other rarer diseases are also present in the literature.*

- Chemical insults from different sources, including many compounds used in agriculture (Genter and Doty, 2019).
- Infections, mostly from viruses, but also from bacteria, fungi, and protozoa (reviewed in Doty, 2019).
- Neurodegeneration (Marin et al., 2018): Parkinson’s disease, multiple sclerosis (Fleiner et al., 2010; Goverover et al., 2020), Alzheimer’s disease, and dementia (Lang et al., 2006).
- Neuropsychiatric illnesses: psychosis (Catalan et al., 2021), anorexia nervosa (Mai et al., 2020), and depression (Athanassi et al., 2021)-noteworthy, a long-known model of depression is the olfactory-bulbectomized rodent, in which the increase of pro-inflammatory cytokines may alter monoamine neurotransmission (Leonard and Song, 2002).
- Obesity (Holinski et al., 2015), metabolic syndrome-type 2 diabetes (Rasmussen et al., 2018; Zhang et al., 2019; Catamo et al., 2021; Faour et al., 2022) and to a lesser extent type 1 diabetes (Falkowski et al., 2020).
- Cachexia, not only in cancer patients (Hutton et al., 2007; Zhou et al., 2017), anorexia of the elderly (Wysokiński et al., 2015).
- Lung cancer (Spencer et al., 2021).
- Metastatic cancer (also not undergoing chemotherapy) (Landis et al., 2004; Spotten et al., 2017).
- Different autoimmune disorders, like fibromyalgia (Amital et al., 2014) and systemic lupus erythematosus (Bombini et al., 2018). A link between smell impairment and autoimmune dysregulation has been devised also for neuropsychiatric disorders (Moscavitch et al., 2009).
- Coronary heart disease is associated with olfactory dysfunction (Seubert et al., 2017), cardiovascular disease in general is more likely associated with olfactory dysfunction in men (Roh et al., 2021), while alteration of smell and taste are prospectively associated with a larger increase in blood pressure (Liu et al., 2018). It may be recalled that epithelial sodium channels participate in salty taste transduction but also in blood pressure regulation, like the olfactory receptors located in the kidney.
- Hemodialyzed, peritoneal dialysis patients or untreated chronic kidney disease patients (Griep et al., 1997; Nigwekar et al., 2017; Robles-Osorio et al., 2020; Iacono et al., 2021).
- Liver disease (Landis et al., 2004) and cirrhosis (Heiser et al., 2018).
- Inflammatory bowel diseases (Steinbach et al., 2013), including Crohn’s disease and ulcerative colitis. The latter patients show also increased peripheral serotonin levels (Sikander et al., 2015).
- Taste thresholds are altered in COPD (Chapman-Novakofski et al., 1999).
- Cystic fibrosis (Di Lullo et al., 2020).

Of note, some of the above-mentioned diseases that may correlate with taste/olfaction impairment are risk factors for a fatal outcome of the SARS-CoV2 virus infection, for example, obesity (Khan et al., 2020), older age, heart disease, dementia, diabetes, and chronic liver or kidney disease (Singh et al., 2020; Kim et al., 2021; CDC, 2022, and references therein).^1^

With few exceptions, only a minor diagnostic value has been attributed to taste/olfaction alterations. At best, a role has been recognized in modifications of food intake because of changes in food hedonic value. As a matter of fact, this variegated list of supposedly unrelated diseases might suggest that chemosensory impairment is an uninteresting side effect potentially caused by many vaguely defined agents.

Conceivably, it makes sense to reverse this interpretation: alterations of taste and olfaction may signal impairment also of other chemosensory systems, and hence have a causal part in worsening the clinical course of diseases because of (up or down) dysregulation of homeostatic mechanisms, which commonly rely on chemosensory inputs. As an example, it may be worth considering two strikingly opposite diseases, cachexia (which includes increased resting energy expenditure and anorexia) and obesity.

These multifactorial syndromes share common biochemical characteristics. In addition to alteration of food intake, similar metabolic changes are present as insulin resistance, wasting of muscle tissue, altered energy expenditure (in both diseases, elevated or reduced depending on the patient), increased lipolysis, unregulated excess protein catabolism, chronic inflammation (Mantovani et al., 1997; Zwickl et al., 2019), deregulated activation of the innate immune system (Tisdale, 2001; Laviano et al., 2017; Antuna-Puente et al., 2021); in most patients similar multiple endocrine dysfunctions are present and, notably, the elevation of peripheral serotonin levels (Crane et al., 2015; Minami et al., 2003). Both central and peripheral serotonin, from the enterochromaffin cells, appear as a major player in the regulation of metabolism (Yabut et al., 2019). All these parameters are, to a large extent, under control of chemosensory systems. A causal relationship is not yet established, but is worth considering.

Under ordinary life conditions, chemosensory impairment of a single system does not seem to be detrimental for survival. Its malfunctioning presumably is to a large extent compensated by the other chemosensory mechanisms. However, under stress conditions, COVID-19 pneumonia for example, and also altered glucose homeostasis (Bentsen et al., 2019), every chemosensory system may become vital for enabling the brain to organize an efficient functional homeostatic response that could effectively increase the survival rate. As an instructive example, the lateral parabrachial nuclei respond to hypoglycemia and are connected to the hypothalamic ventromedial nucleus and subsequently to the bed nucleus of the stria terminalis (Meek et al., 2016), all areas involved in chemosensation, and part of the central autonomic network that orchestrate the body integrated responses (Benarroch, 1993).

Under this perspective, loss of taste and olfaction may be considered not only as a diagnostic tool but also as a warning signal of the impairment of visceral chemosensory systems. Defective chemosensory information and the subsequent dysregulation of CNS homeostatic responses should be considered an important contributor to the higher death rate of COVID-19 in comparison to other ARDS (Hue et al., 2020). Similarly, impairment of chemical senses may have a crucial role in the above-mentioned non-infectious chronic diseases.

## Clinical Testing of Chemosensory Functions

Smell (both orthonasal and retronasal), taste, and also chemesthesis can be easily tested in patients with different techniques, ranging from psychophysical to electrophysiological (Hummel and Podlesek, 2021), validated in humans (Doty, 2018; Whitcroft and Hummel, 2019). The most used psychophysical tests for olfactory function include the Sniffin’ Sticks (Hummel et al., 1997, 2007) and the University of Pennsylvania Smell Inventory Test – UPSIT (Doty et al., 1984) and its cross-cultural version (Doty et al., 1996). More recent tests include the food-associated olfactory test (Denzer-Lippmann et al., 2017) and the culturally independent retronasal test of olfactory function (Croy et al., 2014b). Taste is usually tested with taste strips (Landis et al., 2009; Doty et al., 2021). Psychophysical testing is implemented for trigeminal functions, also in an automated way (Hummel et al., 2016; Huart et al., 2019). The central processing of chemosensory stimuli has been investigated with chemosensory event-related potentials for olfactory and trigeminal stimuli (Kobal and Hummel, 1988) and with the more recent neuroimaging techniques, including PET and fMRI (Small et al., 2004; Gottfried and Zald, 2005; Barresi et al., 2012; Mantel et al., 2019), also using computerized pulse-ejection systems (Kumazaki et al., 2016; Mazzatenta et al., 2016).

When it comes to visceral chemosensory afferents, non-invasive validated procedures are largely lacking, perhaps because their dysfunctions are generally rated low on the list of clinical symptoms and they are not usually examined in ordinary clinical practice.

Procedures that may evaluate (not exclusively) carotid body function are “6 mins walking test” and hypoxic ventilatory response (Grove et al., 1995; Niewinski et al., 2021), which are well-validated, and newer tests are being proposed (Bhattacharyya et al., 2020). In addition, hypoxic pulmonary vasoconstriction and hypoxic or high-altitude bronchoconstriction depend (also) on serotonin release and the activation of pulmonary neuroendocrine cells, for which a specific test is lacking. For enterochromaffin cells and other visceral chemosensory systems, no functional test is available for humans. At present, for visceral chemosensory systems, examination of bioptical or autoptical material is the only available option. Nevertheless, putting chemosensory dysfunction on the clinical spot may help in understanding the patient’s symptoms and the underlying pathophysiology of complex diseases, involving multiple systems’ integrated response.

## Conclusion

The different chemosensory systems should be considered as sharing common characteristics and being involved in the regulation of vital functions, also in humans, under physiological and pathological conditions. Many vital visceral functions are under the control of multiple, apparently overlapping mechanisms, as an example, oxygen partial pressure is measured in the plasma by carotid body cells, in bronchial air by pulmonary neuroendocrine cells, in extracellular fluid by yet unknown tissue receptors, while oxygen binding to hemoglobin is sensed by the aortic bodies. Conversely, none of these sensory systems are actually redundant, each measuring the same parameter under different conditions, fostering the proper functional adaptive responses. Furthermore, these chemosensory systems are not specific for respiratory gases, they measure also different chemical parameters acting as polymodal sensors. Further examples may be quoted from blood pressure control, glucose control, and food intake. Apparently, all these functions rely on a set of chemosensory inputs that are necessary for the brain to properly implement a coordinated homeostatic response.

## Author Contributions

AC and CM-C conceived and wrote the manuscript and approved the submitted version.

## Conflict of Interest

The authors declare that the research was conducted in the absence of any commercial or financial relationships that could be construed as a potential conflict of interest.

## Publisher’s Note

All claims expressed in this article are solely those of the authors and do not necessarily represent those of their affiliated organizations, or those of the publisher, the editors and the reviewers. Any product that may be evaluated in this article, or claim that may be made by its manufacturer, is not guaranteed or endorsed by the publisher.

## Footnotes

1
https://www.cdc.gov/coronavirus/2019-ncov/need-extra-precautions/people-with-medical-conditions.html
